# Predicting Depression Risk in Patients With Cancer Using Multimodal Data: Algorithm Development Study

**DOI:** 10.2196/51925

**Published:** 2024-01-18

**Authors:** Anne de Hond, Marieke van Buchem, Claudio Fanconi, Mohana Roy, Douglas Blayney, Ilse Kant, Ewout Steyerberg, Tina Hernandez-Boussard

**Affiliations:** 1 Clinical AI Implementation and Research Lab Leiden University Medical Centre Leiden Netherlands; 2 Department of Biomedical Data Sciences Leiden University Medical Centre Leiden Netherlands; 3 Department of Medicine (Biomedical Informatics) Stanford Medicine Stanford University Stanford, CA United States; 4 Department of Electrical Engineering and Information Technology ETH Zürich Zürich Switzerland; 5 Department of Medical Oncology Stanford Medicine Stanford University Stanford, CA United States; 6 Department of Digital Health University Medical Centre Utrecht Utrecht Netherlands; 7 Department of Biomedical Data Science Stanford University Stanford, CA United States; 8 Department of Epidemiology & Population Health (by courtesy) Stanford University Stanford, CA United States

**Keywords:** natural language processing, machine learning, artificial intelligence, oncology, depression, clinical decision support, decision support, cancer, patients with cancer, chemotherapy, mental health, prediction model, depression risk, cancer treatment, radiotherapy, diagnosis, validation, cancer care, care

## Abstract

**Background:**

Patients with cancer starting systemic treatment programs, such as chemotherapy, often develop depression. A prediction model may assist physicians and health care workers in the early identification of these vulnerable patients.

**Objective:**

This study aimed to develop a prediction model for depression risk within the first month of cancer treatment.

**Methods:**

We included 16,159 patients diagnosed with cancer starting chemo- or radiotherapy treatment between 2008 and 2021. Machine learning models (eg, least absolute shrinkage and selection operator [LASSO] logistic regression) and natural language processing models (Bidirectional Encoder Representations from Transformers [BERT]) were used to develop multimodal prediction models using both electronic health record data and unstructured text (patient emails and clinician notes). Model performance was assessed in an independent test set (n=5387, 33%) using area under the receiver operating characteristic curve (AUROC), calibration curves, and decision curve analysis to assess initial clinical impact use.

**Results:**

Among 16,159 patients, 437 (2.7%) received a depression diagnosis within the first month of treatment. The LASSO logistic regression models based on the structured data (AUROC 0.74, 95% CI 0.71-0.78) and structured data with email classification scores (AUROC 0.74, 95% CI 0.71-0.78) had the best discriminative performance. The BERT models based on clinician notes and structured data with email classification scores had AUROCs around 0.71. The logistic regression model based on email classification scores alone performed poorly (AUROC 0.54, 95% CI 0.52-0.56), and the model based solely on clinician notes had the worst performance (AUROC 0.50, 95% CI 0.49-0.52). Calibration was good for the logistic regression models, whereas the BERT models produced overly extreme risk estimates even after recalibration. There was a small range of decision thresholds for which the best-performing model showed promising clinical effectiveness use. The risks were underestimated for female and Black patients.

**Conclusions:**

The results demonstrated the potential and limitations of machine learning and multimodal models for predicting depression risk in patients with cancer. Future research is needed to further validate these models, refine the outcome label and predictors related to mental health, and address biases across subgroups.

## Introduction

### Background

Depression in patients with cancer occurs frequently around diagnosis and treatment and has been negatively associated with a patient’s prognosis, quality of life, and treatment adherence [1-5]. Despite affecting up to 20% of patients with cancer and far exceeding the prevalence in the general population (8.4% in the United States [6]), depression is underdiagnosed and often untreated [1,3,7-9]. Constrained clinician time and a strong focus on anticancer treatment may contribute to the insufficient identification of patients at risk for depression [10-13]. Early detection of depression in patients with cancer may enable timely mental health support to augment the anticancer treatment.

Clinical decision support tools with artificial intelligence (AI) technologies could synthesize the abundance of data collected during treatment to help clinicians identify which patients may need specific attention and steer additional mental health resources to those at high risk. A recent review [14] of AI models developed for depression risk in primary care [15], elderly care [16,17], and social media posts [18-20] highlights how AI tools have the potential for early identification of mental health issues. However, oncology-specific applications are rare, and those that do exist are developed on selected small samples that may not generalize to clinical care settings [21,22]. This leaves a gap in oncological care for mental health.

### Objective

We aimed to develop a prediction model for early identification of patients at risk for depression within the first month of chemo- or radiotherapy treatment. We assessed the relevance of different data modalities for predictive performance in a retrospective cohort study.

## Methods

### Data Source and Patient Population

This retrospective observational study used data from the integration of 3 health care organizations: an academic medical center (AMC), a primary and specialty care alliance (PSC), and a community medical center (CMC). These organizations offer a wide spectrum of specialized and advanced health care services for complex medical conditions, operating in over 600 clinics. The PSC, established in 2011, comprises more than 70 primary and specialty clinics throughout the California Bay Area. The CMC provides a range of inpatient and outpatient services in the Tri-Valley region of East Bay and was acquired by the AMC in 2015. Following the merger and acquisition, all health care settings adopted the same Epic-based electronic health record (EHR; Epic Systems Corporation) system. Patients for the study were identified from a clinical data warehouse that consolidated patient data from the AMC, PSC, and CMC from 2008 to 2021 [23]. The EHR system was initiated in 2005, and by 2008, the data had reached a state of robustness and high quality. The study concluded in 2021 to ensure that all patients who visited the clinic during the extended period were comprehensively captured.

As an integral component of the EHR system, the MyHealth portal and web interface are seamlessly incorporated into the EHR. This integration includes a patient portal, enabling patients to engage with their health care teams through secure email communication. Patient-generated emails were systematically gathered from the MyHealth patient portal. These email exchanges feature structured subject lines, with patients selecting from a predefined set of categories such as “Non-Urgent Medical Question,” “Prescription Question,” “Visit Follow-Up Question,” “Test Results Question,” “Update My Health Information,” “Scheduling Question,” and “Ordered Test Question.” The email body allows for free-text input but is limited to 1000 characters. Importantly, all incoming emails are meticulously triaged to the appropriate members of the patient’s health care team, including clerical, scheduling, clinical, or other team members, who take the necessary actions or provide responses as needed [24].

**Figure 1:**
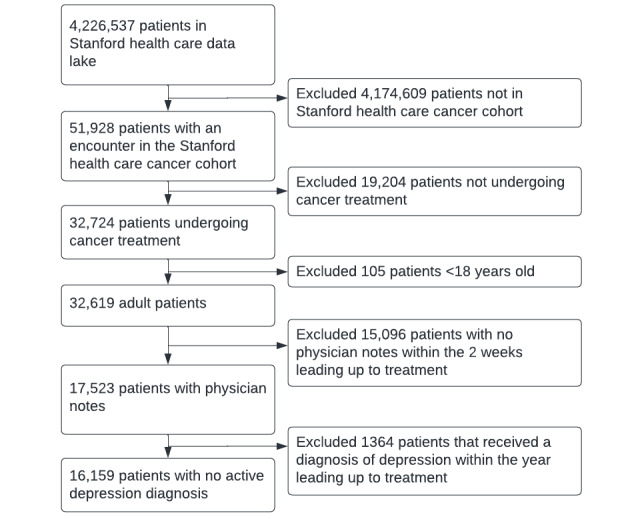
Flowchart of cohort selection.

Adult patients receiving chemo- or radiotherapy treatment were included in the cohort. Given the data-intensive nature of the techniques used [25], our objective was to encompass all eligible patients throughout the entire available period at the time of our analysis. The start of cancer treatment was defined as the first patient encounter that registered chemotherapy (including targeted and immunotherapy) or radiotherapy (“chemotherapy” and “clinical procedure codes” in Multimedia Appendix 1). We excluded patients who did not receive cancer treatment (eg, patients seen for a second opinion only), were younger than 18 years, and had no clinician notes within the 2 weeks leading up to the treatment (Figure 1). We also excluded patients with a depression diagnosis within the year leading up to treatment as we aimed to focus on individuals who are at risk of developing depression during or after their treatment (Figure 1). It was assumed that these patients were already receiving treatment for their depression or at least had additional support offered to them.

### Ethical Considerations

This study was approved by the Stanford institutional review board (#47644). Informed consent was waived for this retrospective study for access to personally identifiable health information as it would not be reasonable, feasible, or practical. The data are housed in the Stanford Nero Computing Platform, which is a highly secure, fully integrated internal research data platform meeting all security standards for high risk and protected health information data. The security is managed and monitored, and the platform is updated and adapted to meet regulatory changes.

### Predictive Outcome

Depression was defined in consultation with oncologist coauthors (DWB and MR) as a depression diagnosis via the *International Classification of Diseases* (ICD)-9 and ICD-10 codes obtained from EHR data (“ICD depression codes” in Multimedia Appendix 1). This end point was chosen as it was the most conservative and has been shown to correlate reasonably well with clinical opinion [26]. Depression risk was predicted within 1 month of cancer treatment. This time window was chosen as depression prevalence is highest during diagnosis and the acute phase of cancer treatment [27].

### Structured Data Predictors

The following variables were obtained from structured EHR fields: sex (male and female), age, insurance status (private, Medicare, Medicaid, and other or not identified), cancer stage (I, II, III, IV, and missing), hospitalized in the previous month (yes or no), 1 or more emergency department visits in the previous month (yes or no), the Charlson comorbidity score [28], and the number of emails sent in the month prior to treatment (none, 1-3, 4, or more) based on a previous study [24]. Insurance status was recoded into 4 comprehensive categories (private, Medicare, Medicaid, and other or not identified). Cancer stage was also recoded to contain the 4 main stages (I, II, III, IV, and missing). Whether or not patients sent emails at night in the previous month was also included as insomnia and depression are intimately related [29]. Binary variables were added indicating whether a patient had previously received a depression diagnosis; depressant medication; or a referral to a psychiatrist, psychologist, or social worker. Finally, race and ethnicity (Hispanic, non-Hispanic Asian, non-Hispanic Black, and non-Hispanic White) was included in one of the sensitivity analyses (see below). The ethnicities “Latino” and “Hispanic” were merged into 1 category (Hispanic). The categorical predictors were converted into dummy variables.

Descriptive statistics were reported in terms of percentages for categorical variables and the mean and SD for continuous variables. We analyzed the cancer and insurance information that was closest to, but preceding, the patient’s start of treatment. We stratified descriptive statistics according to outcome (depression diagnosis or not) and messaging behavior (active email communicator in the past month or not).

### Unstructured Text Predictors

Unstructured text included patient emails with the subject “Non-Urgent Medical Question” sent through a secure patient portal and clinician notes [24].

A Bidirectional Representations from Transformers (BERT) model was trained on a subset of manually labeled emails to classify each email as being “concerning for depression” or not (see the Multimedia Appendix 2 [30-33] for further details on the annotation strategy and model development). Automatically sent emails; copies of previously sent emails; and emails containing questionnaires, appointment requests, and medication refill requests were removed from the set of patient emails. Emails with less than 30 words were removed from the data set. Each email in the final data set was truncated to a maximum token length of 512. This BERT model assigned each patient email a classification score ranging from 0 (not concerning for depression at all) to 1 (most concerning for depression). These email classification scores were summarized at the patient level by calculating the minimum email classification score in the previous month, the maximum score in the previous month, and the mean score in the previous month. These email classification features were then included as structured data in the subsequent model developments.

Clinician notes that were shorter than 100 words or longer than 5000 were removed as these contained erroneous entries or long copies of previous notes, respectively. Notes with mentions of clinical trials, duplicates, and empty notes were also removed. We merged the most recent clinical notes (at most 3) created within the 2 weeks before the start of treatment. The merged notes were decomposed into chunks of at most 25 sequences (to avoid computational issues), each sequence consisting of 256 tokens.

### Model Development

For all models, data were randomly split into the same two-thirds for the train set and one-third for the test set. A total of 6 models were trained to assess the value of multimodal data for this use case.

First, a machine learning (ML) model was developed based on the structured EHR data (model 1), email classification scores (model 2), and the combination of the 2 (model 3). The following ML algorithms were compared for these models: least absolute shrinkage and selection operator (LASSO) logistic regression, a decision tree, random forest, gradient boosting decision trees, *k*-nearest neighbor, and naive Bayes.

LASSO logistic regression is a regularized regression approach, providing both variable selection and shrinkage of regression coefficients. A decision tree is a nonparametric algorithm consisting of a hierarchical tree structure. A random forest combines the predictions of many independently built decision trees into 1 prediction. Gradient boosting decision trees essentially optimize random forest estimation by gradient boosting. The *k*-nearest neighbor algorithm is also nonparametric and uses proximity to previously seen data points to make predictions. Finally, naive Bayes is a generative algorithm that models the distribution of its predictors to make predictions.

The hyperparameters of these models (see Tables S1-S3 in Multimedia Appendix 2) were optimized using Bayesian optimization and 5×10-fold cross-validation. The final ML models were trained on all training data with optimized hyperparameters. The best-performing ML algorithms were the basis for extension with unstructured data.

We trained BERT models based on the clinician notes (model 4), the structured EHR data in combination with the clinician notes (model 5), and the structured EHR data in combination with the email classification scores and the clinician notes (model 6). BERT models are deep learning language models that learn contextual relations between words in a text. Models 5 and 6 made use of a modality-specific deep learning architecture to combine the different data modalities in the modeling process (see Multimedia Appendix 2 for more details) [34]. We used a pretrained DistilBERT model [32] as it required less computation than BERT or ClinicalBERT models [33]. The hyperparameters were tuned on 80% and validated on 20% of the training data. The model parameters of the best-performing epoch on the validation data were chosen for further analyses. Probability estimates were recalibrated via isotonic regression for all models [35].

### Statistical Analysis

Model discrimination was quantified by the area under the receiver operating characteristic curve (AUROC), sensitivity, specificity, positive predictive value (PPV), and negative predictive value (NPV) on the test data. Calibration was assessed through calibration plots, with a calibration intercept and slope as summary performance measures [36]. CIs were obtained via bootstrapping (based on 1000 iterations).

As an initial assessment of clinical usefulness, we performed a decision curve analysis for all 6 models plotting net benefit (NB) across a range of decision probability thresholds [37,38]. NB is defined as the number of true-positive classifications penalized for false-positive classifications [39]. The models have the potential to improve clinical decision-making when they have higher NB than 2 baseline strategies: label all as high risk for developing depression and label none as high risk for developing depression.

### Sensitivity Analysis

Sensitivity analyses were performed on the best-performing model to evaluate the impact of modeling choices on model outcomes. Additional models considered different prediction windows (45 days, 2 months, 3 months, and 6 months after the start of cancer treatment). Moreover, patients dying within these prediction windows are a potential competing risk for patients at risk for depression. We therefore removed these patients from the train and test data and repeated the analyses. Variants of outcome definitions such as predicting a prescription of antidepressant medication and a referral to a psychiatrist, psychologist, or social worker within 1 month of cancer treatment (“antidepressant medication” and “mental health referral” in Multimedia Appendix 1) were considered. These definitions were chosen as they might indicate a patient experiencing depression without being officially diagnosed. We also trained a model on the combined outcome of either receiving a depression diagnosis, antidepressant medication prescription, or referral to a psychiatrist, psychologist, or social worker.

### Fairness Analysis and Including Race and Ethnicity

To identify potential fairness issues for specific demographic groups, AUROC, calibration slope, and intercept were compared across sex and race and ethnicity groups [40]. In addition, race and ethnicity was added as a confounder to assess its effect on subgroup model performance.

### Software, Data, and Reporting

All analyses were performed in Python 3.9.7 (Python Software Foundation). Code is available in a git repository [41]. We followed the MINIMAR reporting guidelines (see Multimedia Appendix 2) [42].

## Results

### Descriptive Statistics

A total of 16,159 patients starting cancer treatment between 2008 and 2021 were included in the analyses, of whom 437 (2.7%) received a diagnosis of depression within 1 month of cancer treatment (Table 1 and Figure 1). The 437 patients receiving a depression diagnosis within 1 month of treatment were, on average, younger, more likely to be female, more likely to be non-Hispanic White, and less likely to be non-Hispanic Asian (Table 1). Moreover, patients with a depression diagnosis made more emergency department visits (Table 1). They were also more likely to have received a previous depression diagnosis more than a year before the start of treatment, a prescription for antidepressant medication, and a mental health referral.

Patients who sent emails (4816/16,159, 29.8%) were more likely to be non-Hispanic White or Asian and be privately insured (Table S4 in Multimedia Appendix 2). On average, they were less likely to be hospitalized but made more emergency department visits 1 month prior to treatment and had a higher Charlson comorbidity score; they were also more likely to have previously received a depression diagnosis, antidepressant medication, and a mental health referral.

**Table 1:** Descriptive statistics of the cancer cohort.

Descriptive statistics				All (N=16,159)		No depression diagnosis within 1 month after onset of treatment (n=15,722, 97.3%)		Depression diagnosis within 1 month after onset of treatment (n=437, 2.7%)
**Demographics**								
	Sex (female), n (%)		8568 (53)		8296 (52.8)^a^		272 (62.2)^a^	
	Age (years), mean (SD)		62 (15)		62 (15)^a^		60 (14)^a^	
**Race and ethnicity, n (%)**								
		Hispanic	1870 (11.6)		1812 (11.5)^a^		58 (13.3)^a^	
		Non-Hispanic Asian	3582 (22.2)		3525 (22.4)^a^		57 (13)^a^	
		Non-Hispanic Black	422 (2.6)		410 (2.6)^a^		<20 (<5)^a^	
		Non-Hispanic White	8864 (54.9)		8583 (54.6)^a^		281 (64.3)^a^	
		Other	1421 (8.8)		1392 (8.9)^a^		29 (6.6)^a^	
**Insurance characteristics, n (%)**								
	Private		8745 (54.1)		8496 (54)		249 (57)	
	Medicare		2590 (16)		2514 (16)		76 (17.4)	
	Medicaid		1917 (11.9)		1860 (11.8)		57 (13)	
	Other or not identified		2907 (18)		2852 (18.1)		55 (12.6)	
**Treatment characteristics, mean (SD)**								
	Number of hospitalizations one month prior to treatment		2083 (13)		2016 (13)		67 (15)	
	Number of emergency department visits 1 month prior to treatment		945 (6)		895 (6)^a^		50 (11)^a^	
	Charlson comorbidity score		6.9 (3.8)		6.9 (3.8)		6.9 (3.9)	
**Tumor type, n (%)**								
		Breast	1772 (11)		1739 (11.1)		33 (7.6)	
		Lung	1001 (6.2)		973 (6.2)		28 (6.4)	
		Prostate	777 (4.8)		764 (4.9)		<20 (<5)	
		Colon and rectum	543 (3.4)		525 (3.3)		<20 (<5)	
		Non-Hodgkin lymphoma	535 (3.3)		527 (3.4)		<20 (<5)	
		Other	3459 (21.4)		3364 (21.4)		95 (21.7)	
		Missing	8072 (50)		7830 (49.8)		242 (55.4)	
**Cancer stage, n (%)**								
		Stage I	1492 (9.2)		1466 (9.3)		26 (5.9)	
		Stage II	1499 (9.3)		1468 (9.3)		31 (7.1)	
		Stage III	1329 (8.2)		1294 (8.2)		35 (8)	
		Stage IV	1758 (10.9)		1699 (10.8)		59 (13.5)	
		Missing	10081 (62.4)		9795 (62.3)		286 (65.4)	
**Patient email information (1 month prior to treatment)**								
	Sent 1 or more emails, n (%)		4070 (25.2)		3943 (25.1)		127 (29.1)	
	Email length in words, mean (SD)		49 (35)		49 (35)		49 (35)	
	Sent emails at night, n (%)		308 (1.9)		296 (1.9)		<20 (<5)	
**Mental health history, n (%)**								
	History of depression diagnosis		400 (2.5)		343 (2.2)^a^		57 (13)^a^	
	History of antidepressant medication		2219 (13.7)		2030 (12.9)^a^		189 (43.2)^a^	
	History of mental health referral		2707 (16.8)		2563 (16.3)^a^		144 (33)^a^	

^a^This was tested at the 5% significance level.

### Performance Statistics

The best-performing ML models were based on LASSO logistic regression (Table 2; Tables S1 and S3 in Multimedia Appendix 2). The model based on structured data alone had an AUROC of 0.74 (95% CI 0.71-0.78). The combination of structured data with email classification scores also had an AUROC of 0.74 (95% CI 0.71-0.78), while a model based solely on email classification scores had an AUROC of 0.54 (95% CI 0.52-0.56). At a high level of sensitivity (0.9 at a decision threshold of 1%; Table 3), the PPV of the best-performing model based on structured data was low (0.04; Table 3). At higher decision thresholds (3% and 10%; Table 3), the PPV was increased to 0.07 and 0.17, respectively, but this came at a cost of sensitivity (0.63 and 0.19).

The BERT model based on the clinician notes performed worst and had an AUROC of 0.50 (95% CI 0.49-0.52; Table 2). Combining structured EHR data with clinician notes did improve AUROC performance (0.71, 95% CI 0.68-0.75; Table 2) and so did adding email classification scores (0.70, 95% CI 0.67-0.73; Table 2).

Calibration was acceptable for all ML models. The BERT-based models tended to produce overly extreme risk estimates even after recalibration.

**Figure 2:**
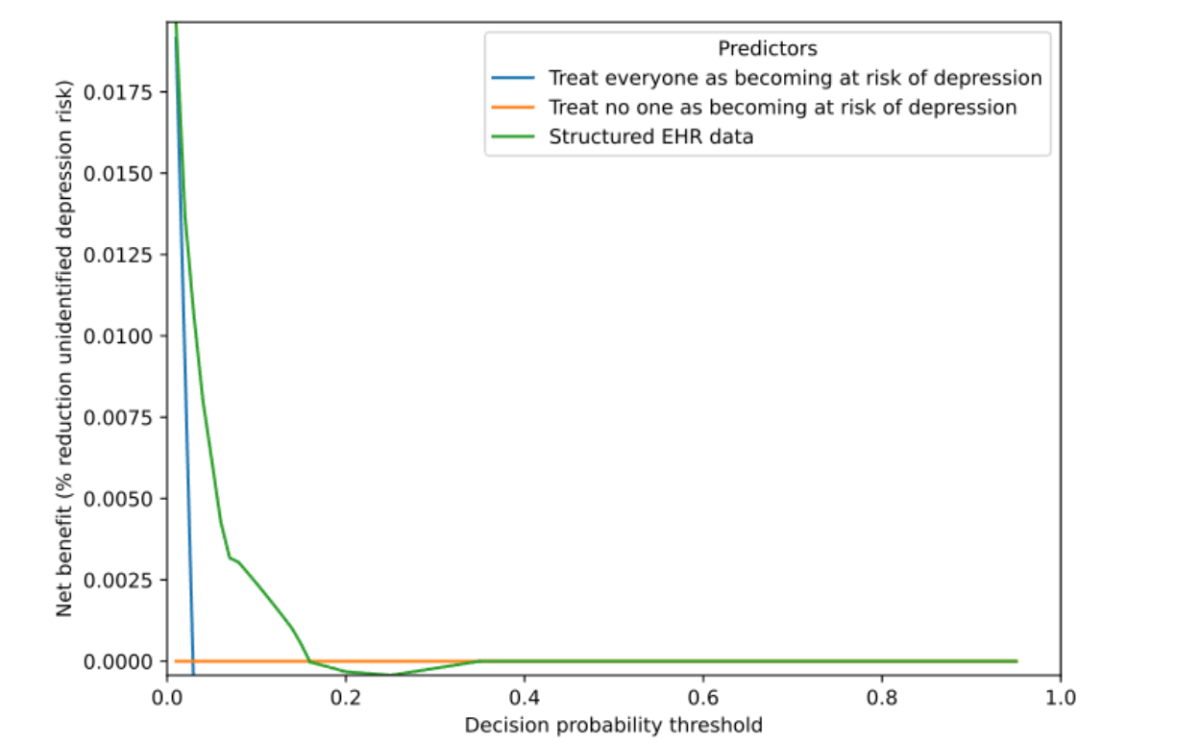
This decision curve analysis (DCA) plots net benefit for the baseline treat all and treat none strategies and the best-performing prediction model (LASSO logistic regression on structured data). EHR: electronic health record; LASSO: least absolute shrinkage and selection operator.

The decision curve analysis showed a small range of decision thresholds for which the best-performing model (LASSO logistic regression based on structured data) had higher NB than the treat all or treat no one strategies (Figure 2). At a decision threshold of 3%, the model with structured EHR data had a NB of 0.01. This represents a net increase of 1 true positive patient at risk for depression per 100 patients without increasing any false positives (at the start of treatment). At a threshold of 10%, the model had a NB of only 0.002, so 2 net true positives per 1000 patients.

**Table 2:** Discrimination and calibration for predicting depression risk within 1 month after the onset of treatment (test data).

Type of data	AUROC^a^ (95% CI)	Calibration intercept (95% CI)	Calibration slope (95% CI)
Structured EHR^b^ data	0.74 (0.71 to 0.78)	0.07 (–0.09 to 0.24)	0.93 (0.77 to 1.09)
Patient emails	0.54 (0.52 to 0.56)	–0.02 (–0.18 to 0.14)	1.0 (0.52 to 1.48)
Structured EHR data and patient emails	0.74 (0.71 to 0.78)	0.07 (–0.09 to 0.24)	0.91 (0.76 to 1.07)
Clinician notes	0.5 (0.49 to 0.52)	–0.05 (–0.21 to 0.11)	0.94 (–1.32 to 3.2)
Structured EHR data and clinician notes	0.71 (0.68 to 0.75)	–0.09 (–0.25 to 0.07)	1.92 (1.57 to 2.28)
Structured EHR data, clinician notes, and patient emails	0.7 (0.67 to 0.73)	–0.16 (–0.32 to –0.0)	2.46 (1.98 to 2.93)

^a^AUROC: area under the receiver operating characteristics curve.

^b^EHR: electronic health record.

**Table 3:** Sensitivity, specificity, PPV^a^, and NPV^b^ at different decision thresholds for predicting depression risk within 1 month after the onset of treatment (test data).

Threshold and analysis		Structured EHR^c^ data	Patient emails	Structured EHR data and patient emails	Clinician notes	Structured EHR data and clinician notes	Structured EHR data, clinician notes, and patient emails
**1%**							
	Sensitivity (n/N)	0.9 (140/156)	1.0 (156/156)	0.87 (136/156)	1.0 (156/156)	1.0 (156/156)	1.0 (156/156)
	Specificity (n/N)	0.35 (1847/5231)	0.0 (0/5231)	0.37 (1915/5231)	0.0 (0/5231)	0.0 (0/5231)	0.0 (0/5231)
	PPV (n/N)	0.04 (140/3524)	0.03 (156/5387)	0.04 (136/3452)	0.03 (156/5387)	0.03 (156/5387)	0.03 (156/5387)
	NPV (n/N)	0.99 (1847/1863)	N/A^d^	0.99 (1915/1935)	N/A	N/A	N/A
**3%**							
	Sensitivity (n/N)	0.63 (98/156)	0.13 (20/156)	0.58 (90/156)	1.0 (156/156)	0.55 (86/156)	0.67 (104/156)
	Specificity (n/N)	0.75 (3912/5231)	0.95 (4962/5231)	0.77 (4032/5231)	0.0 (0/5231)	0.82 (4293/5231)	0.71 (3735/5231)
	PPV (n/N)	0.07 (98/1417)	0.07 (20/289)	0.07 (90/1289)	0.03 (156/5387)	0.08 (86/1024)	0.06 (104/1600)
	NPV (n/N)	0.99 (3912/3970)	0.97 (4962/5098)	0.98 (4032/4098)	N/A	0.98 (4293/4363)	0.99 (3735/3787)
**10%**							
	Sensitivity (n/N)	0.19 (29/156)	0.0 (0/156)	0.19 (30/156)	0.0 (0/156)	0.0 (0/156)	0.0 (0/156)
	Specificity (n/N)	0.97 (5086/5231)	1.0 (5231/5231)	0.97 (5071/5231)	1.0 (5231/5231)	1.0 (5231/5231)	1.0 (5231/5231)
	PPV (n/N)	0.17 (29/174)	N/A	0.16 (30/190)	N/A	N/A	N/A
	NPV (n/N)	0.98 (5086/5213)	0.97 (5231/5387)	0.98 (5071/5197)	0.97 (5231/5387)	0.97 (5231/5387)	0.97 (5231/5387)

^a^PPV: positive predictive value.

^b^NPV: negative predictive value.

^c^EHR: electronic health record.

^d^N/A: not available.

### Sensitivity Analysis

The models predicting depression risk within 45 days (AUROC 0.73, 95% CI 0.69-0.76), 2 months (AUROC 0.73, 95% CI 0.70-0.76), 3 months (AUROC 0.73, 95% CI 0.71-0.76), and 6 months (AUROC 0.72, 95% CI 0.70-0.74) of cancer treatment obtained similar discrimination and calibration compared to the base model predicting depression risk within 1 month (LASSO logistic regression; Table S5 in Multimedia Appendix 2). In the test data, a total of 24 (0.4%) patients died within 1 month after starting treatment. Omitting patients dying within the time frames of interest (1 month-6 months) had no impact on model performance (Table S6 in Multimedia Appendix 2). The model trained to predict depression medication (LASSO logistic regression) also obtained similar discrimination (0.75, 95% CI 0.73-0.78; Table S7 in Multimedia Appendix 2) and calibration compared to the base model predicting depression risk via depression diagnosis. The model trained to predict a referral to a psychiatrist, psychologist, or social worker obtained a lower AUROC of 0.62 (95% CI 0.60-0.64; Table S7 in Multimedia Appendix 2) and comparable calibration.

### Fairness Analysis and Including Race and Ethnicity

The fairness analysis showed that model discrimination was similar for male patients (AUROC 0.73, 95% CI 0.67-0.80; Table S8 in Multimedia Appendix 2) and female patients (AUROC 0.74, 95% CI 0.70-0.78; Table S8 in Multimedia Appendix 2). The calibration plot showed that depression risk was underestimated for female patients and overestimated for male patients (Figure S1 in Multimedia Appendix 2). Discrimination was best for the non-Hispanic Black patients (AUROC 0.92, 95% CI 0.84-0.99; Table S8 in Multimedia Appendix 2), with respect to the non-Hispanic White patients (AUROC 0.74, 95% CI 0.69-0.78; Table S8 in Multimedia Appendix 2) and the non-Hispanic Asian patients (AUROC 0.75, 95% CI 0.63-0.87; Table S8 in Multimedia Appendix 2), and it was worst for Hispanic patients (AUROC 0.71, 95% CI 0.62-0.80; Table S8 in Multimedia Appendix 2). Predictions were underestimated for the non-Hispanic Black patients and overestimated for the non-Hispanic Asian patients (Figure S1 in Multimedia Appendix 2). Adding race and ethnicity as a feature to the best-performing model did not improve model discrimination or calibration (AUROC 0.74, 95% CI 0.71-0.78 vs 0.74, 95% CI 0.70-0.77; Table S9 in Multimedia Appendix 2).

## Discussion

### Principal Findings

This study developed a prediction model to identify patients with cancer at risk for depression within 1 month of chemo- or radiotherapy treatment. We used data from a large comprehensive cancer center with over 16,000 patients. The best-performing models (LASSO logistic regression with structured data with or without patient email classification scores) had reasonable AUROC and calibration. The LASSO logistic regression model with structured data demonstrates a small improvement in NB over the baseline strategy of labeling no one as at risk for depression. Multimodal BERT models (trained on structured data and unstructured text) did not perform better than the best-performing ML model trained solely on structured data.

To date, depression in patients with cancer is underdiagnosed, and studies show that patients with depression are up to 3 times more likely to be noncompliant with medical treatment recommendations [3,43,44]. Treatment adherence is a high priority, given the evidence demonstrating statistically significant associations between treatment nonadherence and patient outcomes, including cancer progression, low-value health care use, and worse survival [45-48]. Therefore, an AI model—which flags patients at risk for depression with minimal clinical input and workflow disruption—is needed at the point of care to prompt clinicians to intervene early and improve patient well-being and anticancer outcomes.

This model may be used in preparation for clinical consultations to more efficiently use the limited time allotted to oncologist-patient interaction to facilitate any needed additional mental health support. By harnessing a combination of structured EHR data and unstructured text data from patient emails and clinician notes, the tool can offer a comprehensive assessment of a patient’s depression risk and help synthesize this information at point of care for the provider. With the ability to establish personalized risk assessments, determine clinical use thresholds, and address potential biases in risk assessment, a clinical decision support tool developed from this work has the potential to significantly enhance the quality of care and mental health outcomes for these vulnerable patients. As the study recognizes the need for ongoing validation, refinement, and bias mitigation, it underscores the dynamic and adaptable nature of this tool in improving cancer care and treatment adherence. This tool can be a valuable addition to the health care system, ultimately improving mental health outcomes and treatment adherence for these vulnerable patients.

The created model has good performance, although our label (receiving a depression diagnosis) depends heavily upon the accurate recognition of depression by the care team. The model’s clinical usefulness depends on the acceptability of the test trade-off. The best-performing model had a high false-positive rate at high levels of sensitivity, and the decision curve analysis showed a test trade-off of 100 assessments for 1 additional true positive patient at a decision threshold of 3%. If these assessments can be done nearly for free (eg, a quick check during a patient visit) and if we already miss all future depressions, then this small improvement may be welcome, although this warrants further validation and testing in the clinical environment. The high false-positive rate and small NB of the best-performing model are likely affected by the moderate discrimination and low event rate [49]. In future developments, the NB may be increased by focusing on improving the labeling of the outcome variable. In addition, richer input data not available to us at the time of analysis could improve model discrimination, like information on lifestyle habits, self-reported mental health assessments, and clinical and pathological factors.

As depression presents differently across sex, race, and ethnicity [50-52], algorithmic fairness forms an important concern when predicting depression risk. We found discrepant model calibration across race, ethnicity, and sex even when controlling for race, ethnicity, and sex in the model. These results align with previous findings that showed poor calibration for minority groups [53,54] and stress the importance of algorithmic fairness assessment in the depression domain. The differences in calibration may be caused by different (recorded) depression rates among groups. This could result in a disproportionate number of missed patients in need of additional mental health resources in specific groups. For example, female and non-Hispanic Black patients might consistently receive a lower predicted risk score than their actual risk. A next step could be to apply bias mitigation techniques for in- or postprocessing during model development, like threshold selection and recalibration within specific groups [55]. Moreover, more diverse data may be collected to adequately capture the differences in symptomatology between different groups. For example, we may include appetite disturbances that are reported more by women and comorbid alcohol and substance abuse that are reported more by men [50].

We also found discrepant model discrimination across race and ethnicity, with the highest AUROC for the non-Hispanic Black group. These findings diverge from the literature, where the AUROC of the minority groups is usually lower compared to the majority group [56]. However, caution is needed when interpreting this finding, due to the very low number of positive cases in this group (less than 20). More data should be collected to better investigate these differences.

The models based solely on text information (patient emails and clinician notes) performed on par with a random coin toss. This implies that the signal-to-noise ratio in this type of data may be too low to be of prognostic value for this specific use case. This might be particularly true for patient emails, where the frequency of the emails varied widely between patients. However, it is important to note that unstructured text, such as patient emails and clinician notes, can potentially provide valuable information that is not captured in structured data. Therefore, multimodal models that incorporate both structured and unstructured data have the potential to improve clinical predictions. Increasing and regularizing the frequency of digital contact between patient and clinician may aid future research on multimodal models in this field, for example, through digital systems for monitoring patient-reported outcomes [57,58]. Digital communication with the aid of chat robots such as ChatGPT [59] provides further direction to better capture patients’ mental health status. This finding also implies that structured data contains strong predictors for depression risk, for example, a history of depression or mental illness, which is well established in the literature and should be considered for future model developments [60-62].

### Limitations

This study had limitations. First, we used the ICD codes for depression diagnosis as indicators of depression risk. This provided a clear and detectable label for our outcome event in the EHR. However, not all patients experiencing depression will receive a coded depression diagnosis with a related ICD code as underdiagnosis is a common problem [3,9]. It is possible that depression may have been diagnosed elsewhere and not recorded in our EHR, that depressive symptoms may have existed and not been recorded or ignored by the oncology-focused clinicians, or that the patient did not express their depressive symptoms to their oncology-focused clinician. In addition, some inconsistencies persisted between the ICD-9 and ICD-10 codes, with the ICD-10 codes including depression associated with bipolar disorder. This may have compromised the accuracy of our predictive models in this exploratory study and should be considered for future research.

Moreover, changing the outcome of interest to either antidepressant medication or a referral to a psychiatrist, psychologist, or social worker did not change the accuracy of the predictive models. An explanation might be that patients with depression are often treated with antidepressants by primary care doctors. For antidepressant medication, it is important to note that there may have been overascertainment as this medication is also used to treat more severe and chronic forms of anxiety. This should be considered when interpreting our results and warrants further study.

Second, the modeling approach was focused on a point-of-care solution, meaning we used clinically meaningful end points (eg, 1 month after starting cancer treatment) and used a diverse patient population. Although this provides the potential for broad application across multiple cancer types, the diversity in cancer types and cancer stages might have introduced noise and impacted model performance.

Third, we used cut-off values for clinician notes that were too short or too long to keep the modeling computationally feasible. This may have led to information loss. Future research may investigate ways of retaining this information when preprocessing texts. Finally, we used data from a single integrated health system for model development, albeit comprised of 3 sites (academic hospital, community hospital, and community practice network). As the cultural background of patients and some data are specific to this health system, our results may not generalize to other populations. Further validation on data sets with different demographics and examination of the mechanisms driving potential biases are needed.

### Conclusions

This study demonstrated the potential and limitations of using structured and unstructured text data for predicting depression risk in patients with cancer using a variety of ML and multimodal models. After further validation and mitigating biases across subgroups, these models have the potential to improve patient outcomes by alerting clinicians of the possible need to escalate support among this vulnerable patient population. Future studies might improve the prediction of depression risk in patients with cancer by refining the outcome label, expanding the predictors related to mental health, and devoting part of the digital patient communication to mental health aspects.

## Appendix

Multimedia Appendix 1 Tabular metadata appendix.

Multimedia Appendix 2 Supplemental methods and results.

## Abbreviations

AI: artificial intelligence
AMC: academic medical center
AUROC: area under the receiver operating characteristic curve
BERT: Bidirectional Encoder Representations from Transformers
CMC: community medical center
EHR: electronic health record
ICD: International Classification of Diseases
LASSO: least absolute shrinkage and selection operator
ML: machine learning
NB: net benefit
NPV: negative predictive value
PPV: positive predictive value
PSC: primary and specialty care alliance

## Data Availability

The data sets generated and analyzed during this study are not publicly available due to the protected nature of the patient data. Requests to access these data sets should be directed to boussard@stanford.edu.
