# Supplementary material for: Predicting Depression Risk in Patients With Cancer Using Multimodal Data: Algorithm Development Study
Source: JMIR Med Inform. 2024 Jan 18;12:e51925. doi: 10.2196/51925 (PMC10835583; doi:10.2196/51925)
Supplement: Multimedia Appendix 2 [file medinform_v12i1e51925_app2.docx]

**Multimedia Appendix 2**

**SUPPLEMENTARY METHODS**

**Email classification model**

Objective

This model aimed to identify emails that raise concern of depression in cancer patients.

Data source and patient population

Emails from cancer patients were extracted from 2009 onwards. Automatically sent emails, copies of previously sent emails, and emails containing questionnaires, appointment requests and medication refill requests were removed from the set of patient emails. A subset of emails was selected for annotation and further processing. We worked with a subset due to our restricted manual annotation capacity. This subset consisted of randomly sampled emails (50%), emails containing concerning words (40%), and emails containing very concerning words (10%). The word lists used to filter emails for concerning or very concerning content were compiled by a social worker.

Classification outcome and annotation process

The classification outcome was defined as an email being ‘concerning for depression’ or not. The softer label ‘concerning for depression’ was chosen as it is not possible to diagnose depression based on an individual email. An annotation guideline was created in collaboration with four clinicians. Seven annotators manually labelled 6600 patient emails. A small subset of emails was annotated by all annotators to assess the inter annotator agreement.

Model development

A Bidirectional Representations from Transformers (BERT) base model was pretrained on a million scraped posts from the r/Depression subreddit to increase the model’s exposure to vocabulary from a population experiencing depression. r/Depression is the biggest subreddit focusing on depression with more than 900.000 members and millions of posts since 2009. This RedditBERT model was subsequently finetuned on 70% of the emails, validated on 15%, and tested on 15%. The RedditBERT model was trained for twenty epochs employing backpropagation with the ADAM optimizer [30] and finetuned on the binary classification task of identifying concerning emails using the annotated sample of patient emails following [31].

**(Multimodal) prediction model**

Model development

We trained a Bidirectional Encoder Representations from Transformers (DistilBERT) model to predict depression risk within one month after the onset of cancer treatment. We chose the distilBERT model [32] as it requires less computation than the BERT or ClinicalBERT models [33]. Three BERT-based models were developed: one model based on clinician notes, one based on structured data and clinician notes (multimodal), and one based on structured data, patient emails, and clinician notes (multimodal). The word embeddings of the clinician notes and structured data modalities were fused in the last layer of the neural network, by concatenating the features before passing them through a sigmoid function. The hyperparameters were tuned on 80% and validated on 20% of the training data. Backpropagation with the ADAM optimizer [30] was used for ten epochs to train the BERT model. Ten epochs were chosen as the BERT models usually converged after fewer epochs. The learning rate for the linear classifier was set to 10^-4^ as the weights were randomly initialized. For the rest of the transformer encoder, the learning rate was set to 10^-5^, as it required only finetuning. The learning rates were halved after five epochs to boost convergence. Additionally, we applied 10^-3^ weight decay during training to avoid blowing up the values of the model parameters. To make a single risk prediction from multiple chunks comprising a clinical note, we averaged their embeddings generated by the BERT model. The model parameters of the best-performing epoch on the validation data were chosen.

**MINIMAR checklist**

| MINIMAR reporting element | Section and page reported in study |
| --- | --- |
| 1. Study population and setting |  |
| Population | Patients and methods - Data source and patient population |
| Study setting | Patients and methods - Data source and patient population |
| Data source | Patients and methods - Data source and patient population |
| Cohort selection | Patients and methods - Data source and patient population |
| 2. Patient demographic characteristics |  |
| Age | Table 1 |
| Sex | Table 1 |
| Race | Table 1 |
| Ethnicity | Table 1 |
| Socioeconomic status | Table 1 (through provider) |
| 3. Model architecture |  |
| Model output | Results |
| Target user | Introduction |
| Data splitting | Patients and methods - Model development |
| Gold standard | Patients and methods - Predictive outcome |
| Model task | Introduction |
| Model architecture | Patients and methods - Model development |
| Features | Patients and methods - Structured data predictors  Patients and methods - Unstructured text predictors |
| Missingness | Table 1 |
| 4. Model evaluation |  |
| Optimization | Patients and methods - Model development |
| Internal model validation | Patients and methods - Model development |
| External model validation | N/A |
| Transparency | Patients and methods - Software and reporting |

**SUPPLEMENTARY RESULTS**

| **Table S1. Discrimination and calibration for predicting depression risk with structured EHR fields within one month after the onset of treatment (test data)** | | | |
| --- | --- | --- | --- |
| Type of machine learning model | AUC | Calibration intercept | Calibration slope |
| Logistic regression  c(0.01,1) = 0.07 | 0.74 (0.71, 0.78) | 0.07 (-0.09, 0.24) | 0.93 (0.77, 1.09) |
| Decision tree  max. depth[2,15] = 3 | 0.71 (0.67, 0.74) | 0.1 (-0.06, 0.27) | 0.92 (0.75, 1.09) |
| Random forest  max. depth[2,15] = 3  n. estimators[100,200] = 195 | 0.73 (0.69, 0.76) | -0.02 (-0.18, 0.14) | 0.99 (0.82, 1.17) |
| Gradient boosting decision trees  max. depth[2,15] = 2  n. estimators[100,200] = 183 | 0.71 (0.68, 0.75) | 0.08 (-0.09, 0.24) | 0.51 (0.40, 0.63) |
| K-nearest neighbors^*^  n. neighbors[2,15] = 15 | 0.62 (0.59, 0.66) | NA | 0.15 (0.10, 0.19) |
| Naïve Bayes  smoothing(0,1) = 1 | 0.72 (0.68, 0.75) | 0.06 (-0.1, 0.22) | 0.81 (0.64, 0.97) |
| Abbreviations: *AUC* Area Under the Receiver Operating Characteristics Curve  ^*^Estimation of the calibration intercept could not converge. | | | |

| **Table S2. Discrimination and calibration for predicting depression risk with email scores within one month after the onset of treatment (test data)** | | | |
| --- | --- | --- | --- |
| Type of machine learning model | AUC | Calibration intercept | Calibration slope |
| Logistic regression  c(0.01,1) = 0.09 | 0.54 (0.52, 0.56) | -0.02 (-0.18, 0.14) | 1.0 (0.52, 1.48) |
| Decision tree  max. depth[2,15] = 3 | 0.49 (0.46, 0.52) | 0.11 (-0.05, 0.27) | -0.13 (-0.24, -0.02) |
| Random forest  max. depth[2,15] = 4  n. estimators[100,200] = 118 | 0.54 (0.51, 0.57) | 0.09 (-0.07, 0.25) | 0.8 (0.38, 1.21) |
| Gradient boosting decision trees  max. depth[2,15] = 2  n. estimators[100,200] = 117 | 0.53 (0.50, 0.56) | 0.09 (-0.07, 0.26) | 0.05 (-0.05, 0.16) |
| K-nearest neighbors  n. neighbors[2,15] = 15 | 0.52 (0.49, 0.54) | 1.44 (1.25, 1.63) | 0.05 (-0.02, 0.12) |
| Naïve Bayes  smoothing(0,1) = 1 | 0.53 (0.51, 0.56) | 0.09 (-0.07, 0.25) | 0.82 (0.42, 1.22) |
| Abbreviations: *AUC* Area Under the Receiver Operating Characteristics Curve | | | |

| **Table S3. Discrimination and calibration for predicting depression risk with structured EHR fields and email scores within one month after the onset of treatment (test data)** | | | |
| --- | --- | --- | --- |
| Type of machine learning model | AUC | Calibration intercept | Calibration slope |
| Logistic regression  c(0.01,1) = 0.09 | 0.74 (0.71, 0.78) | 0.07 (-0.09, 0.24) | 0.91 (0.76, 1.07) |
| Decision tree  max. depth[2,15] = 3 | 0.71 (0.67, 0.74) | 0.1 (-0.06, 0.26) | 0.89 (0.72, 1.05) |
| Random forest  max. depth[2,15] = 4  n. estimators[100,200] = 118 | 0.73 (0.7, 0.77) | 0.04 (-0.13, 0.2) | 0.93 (0.77, 1.09) |
| Gradient boosting decision trees  max. depth[2,15] = 2  n. estimators[100,200] = 117 | 0.7 (0.66, 0.74) | 0.13 (-0.03, 0.3) | 0.47 (0.36, 0.58) |
| K-nearest neighbors^*^  n. neighbors[2,15] = 15 | 0.62 (0.58, 0.65) | NA | 0.14 (0.09, 0.19) |
| Naïve Bayes  smoothing(0,1) = 1 | 0.73 (0.69, 0.76) | 0.06 (-0.1, 0.22) | 0.88 (0.71, 1.05) |
| Abbreviations: *AUC* Area Under the Receiver Operating Characteristics Curve  ^*^Estimation of the calibration intercept could not converge. | | | |

| **Table S4. Descriptive statistics cancer cohort stratified by patients sending emails or not** | | | |
| --- | --- | --- | --- |
|  | All | Did not sent emails in past month | Sent emails in past month |
| **Demographics** |  |  |  |
| N | 16159 (100) | 11343 (70) | 4816 (30) |
| Sex (female), N(%) | 8568 (53) | 5973 (53) | 2595 (54) |
| Age, mean (std) | 62 (15) | 62 (15) | 62 (14) |
| Race/Ethnicity |  |  |  |
| *Non-Hispanic White (%)* | 8864 (55) | 6117 (54)* | 2747 (57)* |
| *Hispanic (%)* | 1870 (12) | 1451 (13)* | 419 (9)* |
| *Non-Hispanic Asian (%)* | 3582 (22) | 2426 (21)* | 1156 (24)* |
| *Non-Hispanic Black (%)* | 422 (3) | 334 (3)* | 88 (2)* |
| *Other (%)* | 1421 (9) | 1015 (9)* | 406 (8)* |
|  |  |  |  |
| **Insurance and provider characteristics** |  |  |  |
| Insurance (%) |  |  |  |
| *Private* | 8745 (54) | 5837 (51)* | 2908 (60)* |
| *Medicare* | 2590 (16) | 1843 (16)* | 747 (16)* |
| *Medicaid* | 1917 (12) | 1479 (13)* | 438 (9)* |
| *Other/Not identified* | 2907 (18) | 2184 (19)* | 723 (15)* |
|  |  |  |  |
| **Treatment characteristics** |  |  |  |
| Number of hospitalizations one month prior to treatment, mean (std) | 2083 (13) | 1590 (14)* | 493 (10)* |
| Number of ED visits one month prior to treatment, mean (std) | 945 (6) | 612 (5)* | 333 (7)* |
| Charlson comorbidity score, mean (std) | 6.9 (3.8) | 6.8 (3.7)* | 7.2 (3.8)* |
| Tumor type (%) |  |  |  |
| *Breast* | 1772 (11) | 979 (9)* | 793 (16)* |
| *Lung* | 1001 (6) | 646 (6)* | 355 (7)* |
| *Prostate* | 777 (5) | 455 (4)* | 322 (7)* |
| *Colon and rectum* | 543 (3) | 399 (4)* | 144 (3)* |
| *(Non-)Hodgkin Lymphoma* | 535 (3) | 341 (3)* | 194 (4)* |
| *Other* | 3459 (21) | 2035 (18)* | 1424 (30)* |
| *Missing* | 8072 (50) | 6488 (57)* | 1584 (33)* |
| Cancer stage |  |  |  |
| *Stage I (%)* | 1492 (9) | 789 (7)* | 703 (15)* |
| *Stage II (%)* | 1499 (9) | 901 (8)* | 598 (12)* |
| *Stage III (%)* | 1329 (8) | 864 (8)* | 465 (10)* |
| *Stage IV (%)* | 1758 (11) | 1121 (10)* | 637 (13)* |
| *Missing (%)* | 10081 (62) | 7668 (68)* | 2413 (50)* |
|  |  |  |  |
| **Mental health history** | | | |
| History of depression diagnosis | 400 (2) | 249 (2)* | 151 (3)* |
| History of anti-depressant medication | 2219 (14) | 1453 (13)* | 766 (16)* |
| History of mental health referral | 2707 (17) | 1604 (14)* | 1103 (23)* |

| **Table S5. Discrimination and calibration for predicting depression risk with structured EHR fields within one month, 45 days, 2 months, 3 months, and 6 months after the onset of treatment (test data)** | | | |
| --- | --- | --- | --- |
| Prediction window | AUC | Calibration intercept | Calibration slope |
| one month | 0.74 (0.71, 0.78) | 0.07 (-0.09, 0.24) | 0.91 (0.76, 1.07) |
| 45 days | 0.73 (0.69, 0.76) | 0.1 (-0.04, 0.24) | 0.95 (0.8, 1.1) |
| two months | 0.73 (0.7, 0.76) | 0.07 (-0.06, 0.2) | 1.02 (0.87, 1.17) |
| three months | 0.73 (0.71, 0.76) | 0.14 (0.02, 0.26) | 1.02 (0.88, 1.15) |
| six months | 0.72 (0.7, 0.74) | 0.08 (-0.02, 0.18) | 0.97 (0.86, 1.09) |
| Abbreviations: *AUC* Area Under the Receiver Operating Characteristics Curve | | | |

| **Table S6. Discrimination and calibration for predicting depression risk with structured EHR fields within one month, 45 days, 2 months, 3 months, and 6 months after the onset of treatment without patients that died in the respective time windows (test data)** | | | |
| --- | --- | --- | --- |
| Prediction window | AUC | Calibration intercept | Calibration slope |
| one month (24 patients died, 0.4%) | 0.74 (0.7, 0.78) | 0.09 (-0.07, 0.25) | 0.93 (0.77, 1.09) |
| 45 days (43 patients died, 0.8%) | 0.73 (0.69, 0.76) | 0.1 (-0.04, 0.25) | 0.96 (0.81, 1.11) |
| two months (59 patients died, 1.1%) | 0.72 (0.69, 0.75) | 0.07 (-0.07, 0.2) | 1.0 (0.85, 1.15) |
| three months (85 patients died, 1.6%) | 0.73 (0.71, 0.76) | 0.14 (0.02, 0.26) | 1.01 (0.88, 1.14) |
| six months (180 patients died, 3.3%) | 0.72 (0.7, 0.74) | 0.11 (0.01, 0.21) | 0.94 (0.82, 1.05) |
| Abbreviations: *AUC* Area Under the Receiver Operating Characteristics Curve | | | |

| **Table S7. Discrimination and calibration for predicting depression risk through depression diagnosis, depression medication, and referral for mental health with structured EHR fields within one month after the onset of treatment (test data)** | | | |
| --- | --- | --- | --- |
| Label | AUC | Calibration intercept | Calibration slope |
| Depression diagnosis | 0.74 (0.71, 0.78) | 0.07 (-0.09, 0.24) | 0.93 (0.77, 1.09) |
| Depression medication | 0.75 (0.73, 0.78) | 0.05 (-0.06, 0.16) | 0.93 (0.83, 1.02) |
| Referral for mental health | 0.62 (0.6, 0.64) | -0.06 (-0.16, 0.03) | 0.84 (0.66, 1.02) |
| Abbreviations: *AUC* Area Under the Receiver Operating Characteristics Curve | | | |

| **Table S8. Discrimination assessment for predicting depression risk with structured EHR fields within one month after the onset of treatment across sex and race/ethnicity (test data)** | |
| --- | --- |
| Subgroup | AUC |
| Male | 0.73 (0.67, 0.8) |
| Female | 0.74 (0.7, 0.78) |
| Non-Hispanic White | 0.74 (0.69, 0.78) |
| Non-Hispanic Asian | 0.75 (0.63, 0.87) |
| Hispanic | 0.71 (0.62, 0.8) |
| Non-Hispanic Black | 0.92 (0.84, 0.99) |
| Abbreviations: *AUC* Area Under the Receiver Operating Characteristics Curve | |

| **Table S9. Effect of including race on discrimination and calibration for predicting depression risk with structured EHR fields within one month after the onset of treatment (test data)** | | | | | |
| --- | --- | --- | --- | --- | --- |
| Including race | AUC | | Calibration intercept | Calibration slope | |
| Without race | 0.74 (0.71, 0.78) | | 0.07 (-0.09, 0.24) | 0.93 (0.77, 1.09) | |
| With race | 0.74 (0.7, 0.77) | | 0.07 (-0.09, 0.24) | 0.88 (0.73, 1.04) | |
| Abbreviations: *AUC* Area Under the Receiver Operating Characteristics Curve | | | | | |
| a)   | | b)   | | |  |
| **Figure S1. Calibration plot for predicting depression risk with structured EHR fields within one month after the onset of treatment across a) sex and b) race (test data)** | | | | |  |
